# Heart Disease

**Published:** 1880-07

**Authors:** 


*An example*—HEART DISEASE.

It is well known that the symptoms of a disease of the heart, and those of the lungs, as well as those of a spinal affection, are so apparently alike in the main, that it requires large medical experience to decide safely and certainly between them; but the exercise requisite in an affection of the lungs would inevitably destroy life if advised for a disease of the heart or spine. In no form of sickness is exercise so immediately and certainly fatal as in heart affections, while the results of active exercise in spinal disease are terrible, literally terrible, not in their immediate effects as involving life, but in the certain penalty of weeks, and months, and weary years of corporeal helplessness, and agonizing, almost ceaseless pain, requiring a thousand times more endurance and a far higher degree of fortitude than marching up to the cannon's mouth in the heat of battle. An affecting instance of this kind came under my notice within a few years past, and I feel sure that a recital of it will be a public benefit, as teaching the importance of taking early competent medical advice in cases of sickness.

I was called to see a young lady on a visit to New York, who was supposed to be in a decline. She was from a neighboring city, an only daughter. She was just entering life, with all the advantages which position, and fortune, and refinement, could bestow. She had a pulse of a hundred and twenty a minute, thirty-six respirations, an incessant cough, debility, such that she could not walk without two assistants. She still lives, a noble monument of heroic endurance and mental energy and worth. Previous to applying to me she had suffered a dozen deaths, in her efforts to take air and exercise on foot, on horse, in carriage; and as often almost as she would take them, she could, on reaching her own door, scarcely prevent herself from shrieking out with an agony of pain. She was encouraged to persevere in these efforts, and, with a daughter's affection for a loving mother, whose solicitude and watchfulness never slept, she did so, until locomotion became impossible. This being a clear case of spinal disease, every step she took, every moment she sat still, aggravated the complaint. The best medical skill in the country has failed to afford her any permanent relief.

